# Investigation of Laminar Convective Heat Transfer for Al_2_O_3_-Water Nanofluids Flowing through a Square Cross-Section Duct with a Constant Heat Flux

**DOI:** 10.3390/ma8085246

**Published:** 2015-08-19

**Authors:** Hsien-Hung Ting, Shuhn-Shyurng Hou

**Affiliations:** Department of Mechanical Engineering, Kun Shan University, Tainan 71070, Taiwan; E-Mail: e056ting@gmail.com

**Keywords:** nanofluid, convective heat transfer, square cross-section duct, laminar flow

## Abstract

The objective of this study is to numerically investigate the convective heat transfer of water-based Al_2_O_3_ nanofluids flowing through a square cross-section duct with a constant heat flux under laminar flow conditions. The effects of nanoparticle concentration and Peclet number on the heat transfer characteristics of Al_2_O_3_-water nanofluids are investigated. The nanoparticle diameter is 25 nm and six particle concentrations (0.2, 0.5, 1, 1.5, 2, and 2.5 vol.%) are considered. The numerical results show that the heat transfer coefficients and Nusselt numbers of Al_2_O_3_-water nanofluids increase with increases in the Peclet number as well as particle volume concentration. The heat transfer coefficient of nanofluids is increased by 25.5% at a particle volume concentration of 2.5% and a Peclet number of 7500 as compared with that of the base fluid (pure water). It is noteworthy that at the same particle volume concentration of 2.5%, the enhancement of the convective heat transfer coefficient of Al_2_O_3_-water nanofluid (25.5%) is much higher than that of the effective thermal conductivity (9.98%). Thus, the enhancement of the convective heat transfer cannot be solely attributed to the enhancement of the effective thermal conductivity. Additionally, the numerical results coincide well with the published experimental data.

## 1. Introduction

Nanofluids, a new class of heat transfer fluids, are liquid suspensions of nanometer-sized particles. Typically, nanofluids contain nanoparticles with sizes of 1 to 100 nm dispersed in a conventional liquid such as water [1,2], ethylene glycol [3], methanol [4], and engine oil [5]. The thermal conductivities of nanofluids with suspended metallic or nonmetallic particles are expected to be significantly higher than those of traditional heat transfer fluids. Accordingly, the suspended nanoparticles can significantly change the transport and thermal properties of a conventional liquid (base fluid). The remarkable enhancement of forced convective heat transfer has been extensively investigated in Cu-water- [6,7,8,9], CuO-water- [10,11], TiO_2_-water- [8,10], Al_2_O_3_-water- [1,2,8], and carbon nanotube-water-based [12] nanofluid thermal systems.

Bianco *et al.* [1] reported an analysis based on the second law of thermodynamics applied to a water-Al_2_O_3_ nanofluid in turbulent convection inside a circular tube subjected to constant wall temperature. It was found that the considered inlet condition influences the different mechanisms and the amount of entropy generation. In particular, at a constant Reynolds number, there is an increase of entropy generation, whereas at a constant mass flow rate or velocity, entropy generation decreases. Later, Bianco *et al.* [2] developed a performance analysis of Al_2_O_3_-water nanofluids by analyzing entropy generation and a performance evaluation criterion based on the first and second law of thermodynamics. Their results demonstrated that with an increase of nanoparticle concentration, not only the Nusselt number increases, but entropy generation and pumping power also increase.

Wen and Ding [13] investigated the convective heat transfer characteristics in Al_2_O_3_-water nanofluid along a copper tube and found that increasing the Reynolds number and volumetric ratio of particles enhanced the heat transfer coefficient. Heris *et al.* [14,15] studied the heat transfer coefficient in Al_2_O_3_-water nanofluid flowing through a duct with a constant wall temperature. It was found that decreasing nanoparticle size and increasing nanoparticle concentration increased the heat transfer coefficient. 

Mirmasoumi and Behzadmehr [16] numerically investigated the effect of the nanoparticle diameter on the convective heat transfer performance of Al_2_O_3_-water nanofluid flowing under a fully developed laminar flow regime. It was shown that decreasing the nanoparticle diameter markedly increased the heat transfer coefficient of the nanofluid; the nanoparticle diameter had no significant effect on the skin friction coefficient.

Shahi *et al.* [17] numerically investigated the performance of the laminar convective heat transfer of Cuo-water nanofluid flowing through a square cavity under a laminar flow regime. It was found that with increasing particle concentration, the average Nusselt number of the nanofluid increased, whereas the bulk temperature of the nanofluid decreased.

Mohammed *et al.* [18,19] numerically investigated the effect of four nanofluids (containing Al_2_O_3_, SiO_2_, Ag, and TiO_2_, respectively) flowing on parallel square and rectangular microchannel heat exchangers. The results showed that with an increase in the Reynolds number, the heat transfer coefficient of the nanofluid increased, whereas the average bulk temperature of the cold fluid decreased.

Recently, flow and heat transfer characteristics of magneto-hydrodynamics nanofluid flow [20,21,22] (such as magnetic Fe_3_O_4_-water nanofluid flow) and heat as well as the mass transfer phenomenon of blood flow of nanofluid have also received attention [23]. In addition to Newtonian nanofluids, the issue of convective heat transfer enhancement using nanoparticles suspended in non-Newtonian fluids has attracted the interest of many researchers. Some studies related to the topic of non-Newtonian fluids can be seen from the list of references [24,25,26].

Much attention has been paid to the heat transfer characteristics of nanofluids. However, few papers have put particular emphasis on the convective heat transfer properties of nanofluids through non-circular ducts. The present study numerically investigates the characteristics of the convective heat transfer of water-based Al_2_O_3_ nanofluids flowing through a square cross-section duct with a constant heat flux under laminar flow conditions. The nanoparticles have a diameter of 25 nm. Six particle concentrations (0.2, 0.5, 1, 1.5, 2, and 2.5 vol.%) are considered. Furthermore, the effect of the Peclet number on the convective heat transfer coefficient is investigated and the results are compared with those of Heris *et al.*’s experimental study [27].

## 2. Mathematical Modeling

### 2.1. Assumptions and Governing Equations

In this numerical study, the single-phase approach for nanofluids is employed [28,29]. It is assumed that the base fluid and nanoparticles are perfectly mixed and thus can be treated as a homogeneous mixture. The flow is laminar and steady-state. Moreover, it is assumed that the fluid phase and solid particles are in thermal equilibrium and move with the same local velocity considering the ultra-fine (25 nm) and low volume fraction (2.5%) of the solid particles. 

The following nonlinear governing equations represent the mathematical formulation of the single-phase model, which include conservation of mass, momentum, and energy for the nanofluid flow inside the square cross-section duct.

Conservation of mass:

(1) $$div\;(\rho_{nf}\vec{V})=0,$$

Conservation of momentum:

(2) $$div\;(\rho_{nf}\vec{V}\vec{V})=-\nabla P+\mu_{nf}\nabla^{2}\vec{V},$$

Conservation of energy:

(3) $$div\text{ (}\rho_{nf}\vec{V}C_{nf}T)=div\;(k_{nf}\nabla T)$$

where $\vec{V}$, *P*, and *T* are respectively the fluid velocity vector, pressure, and temperature; *ρ*, *µ*, *k*, and *C* are the density, dynamic viscosity, thermal conductivity, and specific heat capacity, respectively; subscript *nf* represents a nanofluid property. All fluid properties are calculated at the reference temperature (*i.e.*, the fluid inlet temperature *T_b_*_,*i*_).

### 2.2. Physical Properties of Nanofluid

The physical properties of the nanofluid, including density, thermal conductivity, and viscosity, are defined as follows.

Effective density *ρ_nf_* of the nanofluid:

(4) $$\rho_{nf}=(1-\varphi )\rho_{bf}+\varphi \rho_{p}$$

Effective specific heat capacity *C_nf_* of the nanofluid:

(5) $$C_{nf}=\frac{(1-\varphi ){\left(\rho C\right)}_{bf}+\varphi {\left(\rho C\right)}_{p}}{\rho_{nf}}$$

Effective thermal conductivity $k_{nf}$ of the nanofluid:

Equation (6) [30] is used to calculate the thermal conductivity of the nanofluid.

(6) $$k_{nf}=\frac{k_{p}+2k_{bf}+2(k_{p}-k_{bf}){\left(1+\gamma \right)}^{3}\varphi }{k_{p}+2k_{bf}-2(k_{p}-k_{bf}){\left(1+\gamma \right)}^{3}\varphi }k_{bf}$$

where *γ* is the ratio of the nano-layer thickness to the original particle radius, which is considered to be equal to 0.1.

Effective viscosity *µ_nf_* of the nanofluid:

Equation (7) [27] is employed to calculate the nanofluid viscosity.

(7) $$\mu_{nf}=(1+2.5\varphi )\mu_{bf}$$

The physical properties of Al_2_O_3_ nanoparticles are: thermal conductivity *k_p_* = 46 W/m-K, density $\rho_{p}$ = 3700 kg/m^3^, and specific heat capacity *C* = 880 J/kg-K.

Water-based Al_2_O_3_ nanofluids with various volume fractions (0.2%, 0.5%, 1.0%, 1.5%, 2.0%, and 2.5%) are used as working fluids. In addition, for comparison, water is also employed as the working fluid. The convective heat transfer coefficient is investigated for various Reynolds numbers in the range of 700 < *Re* < 2050. *Re_nf_*, *Pr_nf_*, and *Pe_nf_* are the Reynolds, Prandtl, and Peclet numbers of the nanofluid, respectively, expressed as:

(8) Renf=ρnfU¯Dh μnf

(9) $${\mathrm{Pr}}_{nf}=\frac{C_{\text{nf}}\;\mu_{nf}\;}{k_{nf}}$$

(10) Penf=Renf Prnf=ρnf CnfU¯Dh knf

### 2.3. Boundary Conditions

Boundary conditions are specified as follows.

• (1) At the inlet: profiles of uniform axial velocity $\bar{U}$ and uniform temperature *T_b_*_,*i*_ are used as the inlet velocity and temperature conditions, respectively. That is, *u* = $\bar{U}$, *v* = *w* = 0, and *T* = *T_b_*_,*i*_.
• (2) At the outlet: the fully developed conditions prevail, namely, all axial derivatives are zero, $\frac{\partial u}{\partial x}=\frac{\partial v}{\partial x}=\frac{\partial w}{\partial x}=\frac{\partial T}{\partial x}=0$.
• (3) On the tube wall: no-slip conditions, *u* = *v* = *w* = 0, and constant heat flux (18,500 W/m^2^) are imposed.

### 2.4. Solver

**Figure 1:**
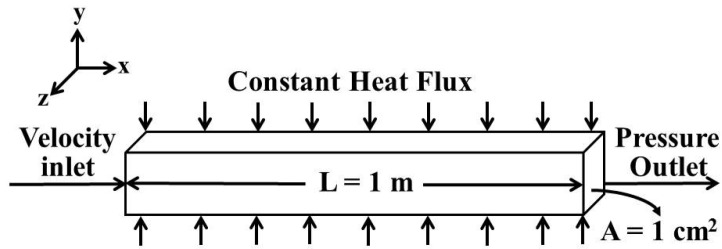
Geometrical configuration used in simulation.

ANSYS FLUENT computational fluid dynamics (CFD) software incorporated with a finite volume method is employed to solve the nonlinear governing equations (Equations (1)–(3)) of laminar force convection heat transfer in a square cross-section duct with a constant heat flux. The control volume-based technique is used to convert a general scalar transport equation into an algebraic equation that can be solved numerically. It consists of: (1) division of the domain into discrete control volumes using a computational grid; (2) integration of the governing equations on the individual control volumes to construct algebraic equations for the discrete dependent variables (“unknowns”) such as velocities, pressure, and temperature; and (3) linearization of the discretized equations and solution of the resultant linear equation system to yield updated values of the dependent variables [31]. Details about the solver algorithms used by ANSYS FLUENT^®^ can be found in reference [31]. Figure 1 shows the geometrical configuration used in the simulation. A 1.0-m-long duct with a square cross-section area of 1 cm^2^, which is exactly the same as that used in Heris *et al.*’s experiment [27], is employed. The GAMBIT (Geometry And Mesh Building Intelligent Tool) [31] model is employed to describe the problem. The model graphs and meshes the spatial domain with a size of (200 × 30 × 30) grids, 200 with length of duct and 30 × 30 with square cross-section area.

The numerical simulation is carried out at various Peclet numbers and particle concentrations (0.2, 0.5, 1.0, 1.5, 2.0, and 2.5 vol.%). The particle diameter is 25 nm. The finite volume formulation is used with the SIMPLE (Semi-Implicit Method for Pressure-Linked Equations) algorithm to solve the discretized equations derived from the partial nonlinear differential equations of the mathematical model. The convection terms of the transport equations are discretized by the second-order hybrid central differences/upwind scheme. During the calculation, the residuals of the algebraic discretized equations, resulting from the spatial integration of the conservation equations over finite control volumes, are monitored. Simulations are considered to be converged when the residuals for all discretized equations are smaller than 10^−6^. Then, the heat transfer coefficient and Nusselt number are respectively calculated using the following equations:

(11) $${\bar{h}}_{nf}=\frac{C_{nf}\rho_{nf}\bar{U}A(T_{b,o}-T_{b,i})}{\pi D_{h}L{\left(T_{w}-T_{b}\right)}_{M}}$$

(12) $${\bar{Nu}}_{nf}=\frac{{\bar{h}}_{nf}D_{h}}{k_{nf}}$$

where ${\bar{h}}_{nf}$ and ${\bar{Nu}}_{nf}$ are the average heat transfer coefficient and Nusselt number of the nanofluid, respectively; *L* is the length of the duct; *D_h_* is the hydraulic diameter of the duct; $\bar{U}$ is the mean velocity of the nanofluid at the inlet; (*T_w_ – T_b_*)*_M_* is the mean temperature difference; *T_b_*_,*i*_ and *T_b_*_,*o*_ are the inlet and outlet bulk temperature of the nanofluid, respectively.

## 3. Results and Discussion

### 3.1. Grid-Independence Analysis 

**Figure 2:**
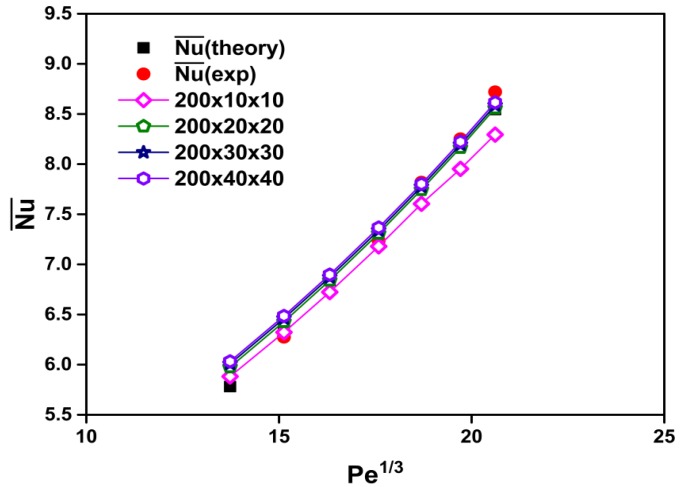
Grid sensitivity testing and comparison among numerical, theoretical [32], and experimental data [27] for the Nusselt number of water.

Initially, to carry out the grid-independence analysis, several non-uniform grids were subjected to an extensive testing procedure. The effects of the number of mesh points on the Nusselt number of water are shown in Figure 2. In this study, the numbers of grid points in the *x*-, *y*-, and *z*-directions are set to 200, 30, and 30, respectively. Finer grid points would not significantly influence the accuracy of the Nusselt number.

### 3.2. Validation

For validation of the CFD simulation, experimental data (Heris *et al.* [27]) and theoretical solutions (Equation (13); Sieder-Tate equation [32]) are used for a comparison in which distilled water (base fluid) is employed as the working fluid. To validate the accuracy and reliability of the present CFD analysis, the calculated results are compared with the experimental data [27] and theoretical solutions [32] for the Nusselt number *versus* the (Peclet number)^1/3^.

(13) Nu=1.86+(RenfPrnfDhL)1/3(μnfμwnf)0.14

where *µ_wnf_* is the nanofluid viscosity at the duct wall temperature.

Figure 2 also shows a comparison among the calculated, experimental [27], and theoretical [32] results in the fully developed laminar regime. It can be seen that the results of the present CFD analysis show good agreement with those of experimental [27] and theoretical [32] studies.

### 3.3. Effects of Peclet Number and Particle Volume Concentrations

**Figure 3:**
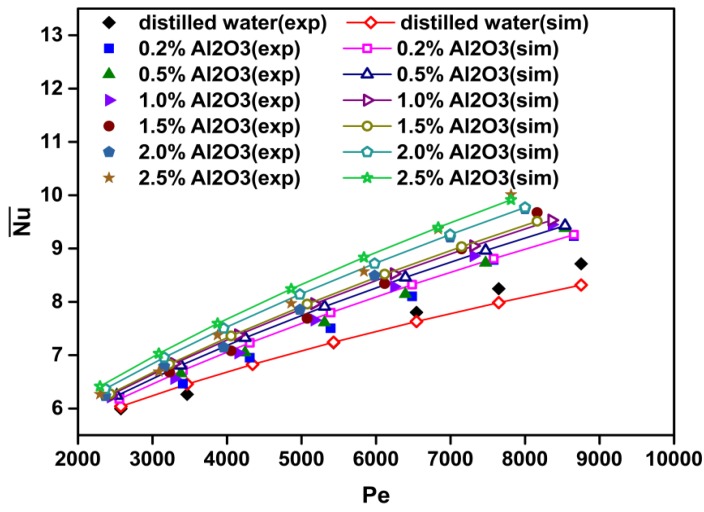
Comparison between numerical and experimental data [27] for Nusselt number *versus* Peclet number at various particle volume concentrations for Al_2_O_3_-water nanofluids.

Figure 3 shows a comparison between numerical and experimental data for the Nusselt number *versus* the Peclet number at various particle volume concentrations for Al_2_O_3_-H_2_O nanofluids. It is found that increasing the particle volume concentration results in a significant increase in the Nusselt number. This is due to an increase in fluid thermal conductivity and an increase of energy exchange rate resulting from the irregular and chaotic motion of ultra-fine particles in the fluid [33]. Additionally, the Peclet number (or Reynolds number) significantly affects heat transfer characteristics. A higher Peclet number (or Reynolds number) corresponds to higher fluid velocity and temperature gradient, which in turn results in a higher value of the Nusselt number. With a constant nanoparticle volume fraction, the increment of *Pe* (or *Re*) with the nanofluid flow rate leads to convective heat transfer enhancement. The better heat transfer enhancement may be caused by better chaotic movement and nanoparticle migration, especially near the duct corner through the flow [27].

**Figure 4:**
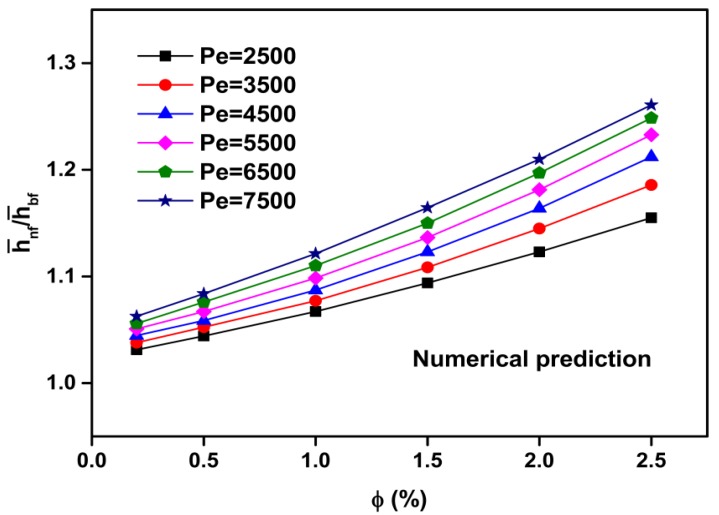
Numerical prediction for ratio of heat transfer coefficient *versus* particle volume concentration at various Peclet numbers for Al_2_O_3_-water nanofluids.

**Figure 5:**
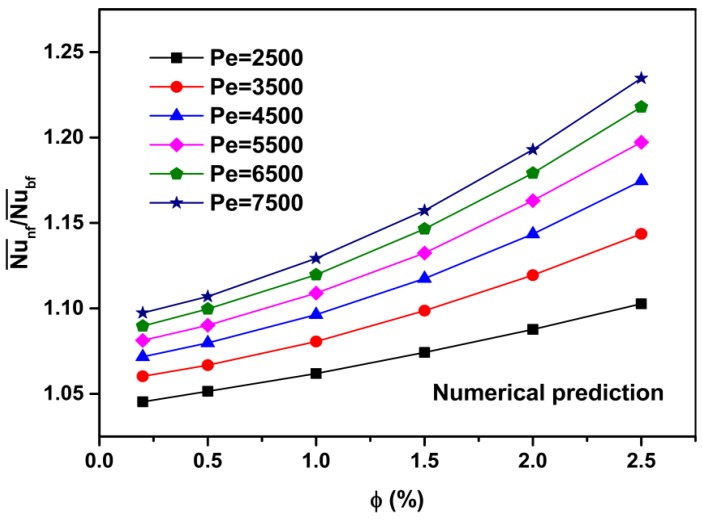
Numerical prediction for ratio of Nusselt number *versus* particle volume concentration at various Peclet numbers for Al_2_O_3_-water nanofluids.

Figure 4 shows the average heat transfer coefficient ratios of the nanofluid to the base fluid (${\bar{h}}_{nf}$/${\bar{h}}_{bf}$) as a function of the Peclet number and particle volume concentration, and Figure 5 shows the average Nusselt number ratios of the nanofluid to the base fluid (${\bar{Nu}}_{nf}$/${\bar{Nu}}_{bf}$) as a function of the Peclet number and particle volume concentration. The calculated results show that the average heat transfer coefficient and Nusselt number of the nanofluids increase with the increasing Peclet number and particle volume concentration. For instance, at *Pe* = 7500, the heat transfer enhancement is about 25.5% for the nanofluid with *ϕ* = 2.5% as compared with that of pure water (Figure 4). Moreover, at Pe = 7500 and *ϕ* = 2.5%, the Nusselt number is increased by about 22.5% compared with that of pure water (Figure 5).

It is interesting to note that for a fixed particle volume concentration of 2.5%, the enhancement of the heat transfer coefficient of Al_2_O_3_-water nanofluid (25.5%) is much higher than that of the effective thermal conductivity (9.98%) predicted by Equation (6). Therefore, the enhancement of the convective heat transfer cannot be solely attributed to the enhancement of the effective thermal conductivity. Similar results were reported by Heris *et al.* [27] and Wen and Ding [34]. Actually, other factors such as dispersion [34], Brownian motion [35], thermophoresis [35], and nanoparticle migration [34,35] may also be responsible for the enhancement of convective heat transfer [27]. Pak and Cho [36] compared the percentage increment in the heat transfer coefficient with that in the effective thermal conductivity of nanofluids and found that the former is greater than the latter. They attributed this to enhanced mixing caused by nanoparticles near the walls. Heyhat and Kowsary [35] investigated the effect of particle migration on heat transfer enhancement and reported that the non-uniform distribution of nanoparticles due to Brownian motion and thermophoresis in nanofluids led to a higher heat transfer coefficient. They proposed that particle migration is an important reason for explaining the further heat transfer enhancement observed in the convective heat transfer of nanofluids.

**Figure 6:**
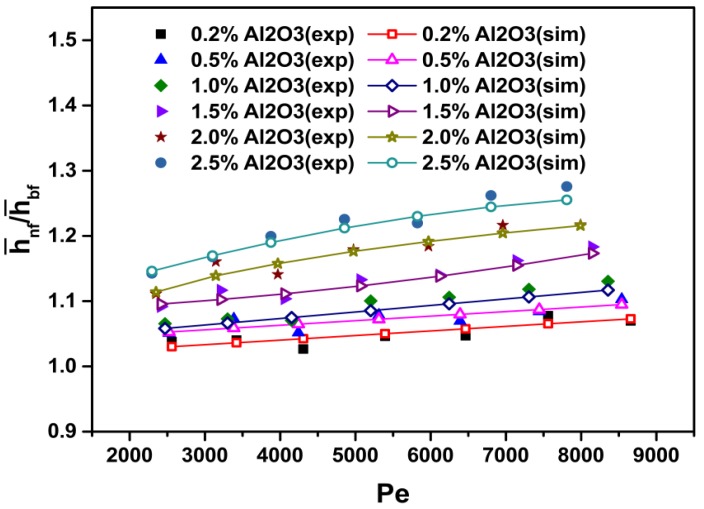
Comparison between numerically predicted and experimentally measured [27] heat transfer coefficient at various particle volume concentrations and *Pe* values for Al_2_O_3_-water nanofluids.

**Figure 7:**
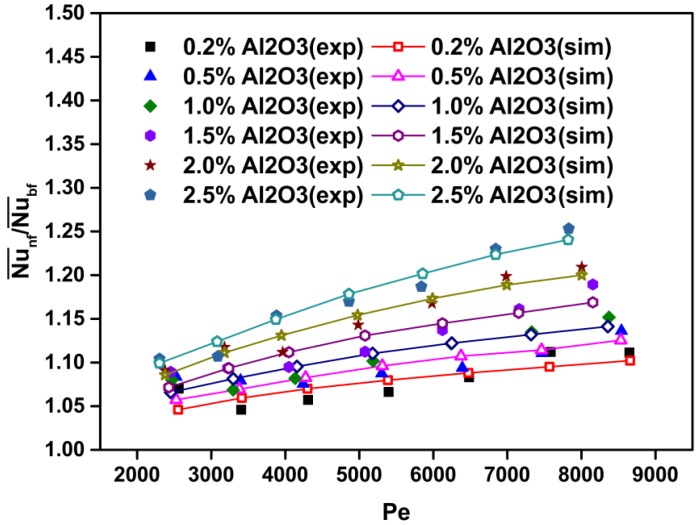
Comparison between numerically predicted and experimentally measured [27] Nusselt number at various particle volume concentrations and *Pe* values for Al_2_O_3_-water nanofluids.

Figure 6 and Figure 7 show a comparison of the average heat transfer coefficient and Nusselt number between the present CFD results and the experimental ones [27], respectively. It is clear that the results of the average heat transfer coefficient and Nusselt number obtained from the simulation coincide well with published experimental data [27]. It is also found that the heat transfer coefficient and Nusselt number increase with the particle volume concentration and Peclet number. The reasons for the heat transfer enhancement are explained above.

**Figure 8:**
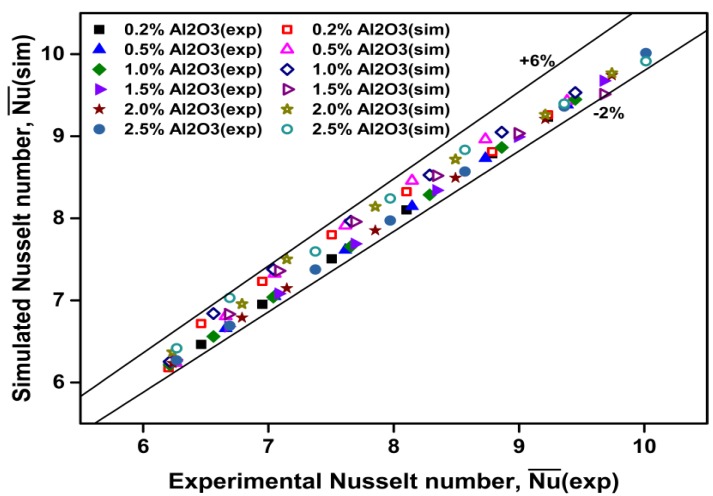
Comparison of measured [27] and predicted Nusselt number for nanofluids.

Figure 8 compares the simulated Nusselt numbers with experimental ones for the water-based Al_2_O_3_ nanofluids. As can be seen, the simulated Nusselt numbers are in good agreement with the values of Heris *et al.*’s experimental data [27]. The discrepancies are in the range of –2% to +6%.

**Figure 9:**
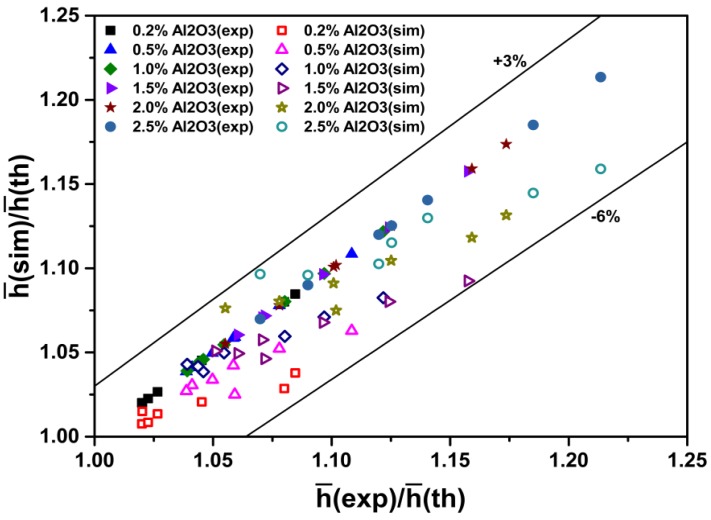
Comparison of ratio of measured [27] and predicted heat transfer coefficient to theoretical heat transfer coefficient for nanofluids.

Figure 9 shows a comparison between ${\bar{h}}_{nf}\text{(sim)}$/${\bar{h}}_{nf}\text{(th)}$ and ${\bar{h}}_{nf}\text{(exp)}$/${\bar{h}}_{nf}\text{(th)}$, where ${\bar{h}}_{nf}\text{(exp)}$ and ${\bar{h}}_{nf}\text{(sim)}$ are experimental [27] and simulated average heat transfer coefficients, respectively, and ${\bar{h}}_{nf}\text{(th)}$is the theoretical convective heat transfer coefficient calculated from the Sieder-Tate equation [32]. ${\bar{h}}_{nf}\text{(sim)}$/${\bar{h}}_{nf}\text{(th)}$ denotes the ratio of the simulated average heat transfer coefficient to the theoretical one calculated from the Sieder-Tate equation, and ${\bar{h}}_{nf}\text{(exp)}$/${\bar{h}}_{nf}\text{(th)}$designates the ratio of the experimental average heat transfer coefficient to the theoretical one calculated from the Sieder-Tate equation. As can be observed, the ratio of the simulated average heat transfer coefficient to the theoretical one is in good agreement with that of the experimental average heat transfer coefficient to the theoretical one. The discrepancies are in the range of –6% to +3%.

**Figure 10:**
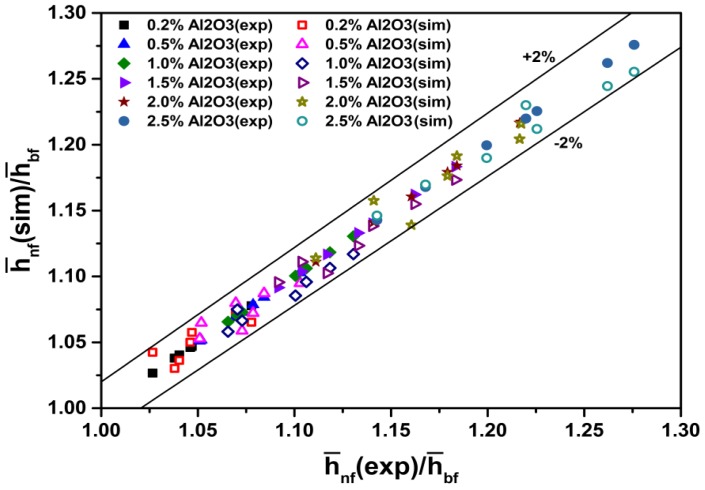
Comparison of ratio of measured [27] and predicted heat transfer coefficient to water heat transfer coefficient for nanofluids.

Figure 10 shows a comparison between ${\bar{h}}_{nf}\text{(sim)}$/${\bar{h}}_{bf}$ and ${\bar{h}}_{nf}\text{(exp)}$/${\bar{h}}_{bf}$. The results show that the ratio of the simulated average convective heat transfer coefficient to that of water coincides well with the ratio of the experimental average convective heat transfer coefficient [27] to that of water. The discrepancies are in the range of –2% to +2%. 

In summary, as discussed previously (Figure 8, Figure 9 and Figure 10), the simulated Nusselt numbers and heat transfer coefficients are in good agreement with the experiment conducted by Heris *et al.* [27]. The discrepancies are within 6%.

**Figure 11:**
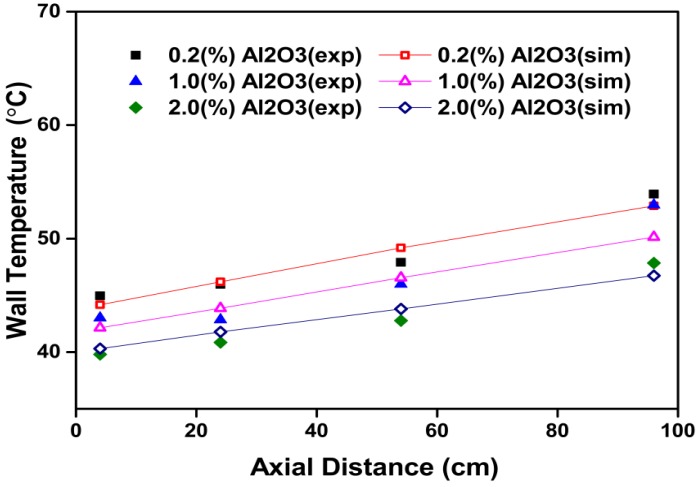
Comparison between numerical and experimental data [27] for wall temperature of square cross-section duct *versus* axial distance at various particle volume concentrations for Al_2_O_3_-water nanofluids.

Figure 11 shows variations of the wall temperature with axial distance at various particle volume concentrations. It can be found that with increasing particle volume concentrations, the cooling rate increases due to an increase in heat transfer enhancement, and in turn the wall temperature decreases.

## 4. Conclusions

The laminar flow-forced convection of Al_2_O_3_-water nanofluids in a square cross-section duct subjected to a constant heat flux was numerically studied. The results show that the heat transfer coefficient and Nusselt number increase with an increasing Peclet number and particle volume concentration. The heat transfer coefficient of Al_2_O_3_-water nanofluid is increased by 25.5% at a particle concentration of 2.5 vol. % compared with that of pure water at *Pe* = 7500. It is noteworthy that, for a fixed particle volume concentration of 2.5%, the enhancement of the convective heat transfer coefficient of Al_2_O_3_-water nanofluid (25.5%) is much higher than that of the effective thermal conductivity (9.98%). Therefore, the enhancement of the convective heat transfer cannot be solely attributed to the enhancement of the effective thermal conductivity. Other mechanisms such as dispersion, Brownian motion, thermophoresis, migration, and the collision intensification of nanoparticles may also be responsible for convective heat transfer enhancement. Moreover, the simulated Nusselt numbers are in good agreement with the values of Heris *et al.*’s experiments [27]. The discrepancies are within 6%. 

## Nomenclature

### Abbreviations

A: Surface area of square cross-section duct (m^2^)
*C*: Specific heat (kJ·kg^–1^·K^–1^)
*D_h_*: Hydraulic diameter (m)
${\bar{h}}_{nf}\text{(exp)}$: Experimental average nanofluid heat transfer coefficient (W·m^–^^2^·K^–^^1^)
${\bar{h}}_{nf}\text{(sim)}$: Simulated average nanofluid heat transfer coefficient (W·m^–^^2^·K^–^^1^)
${\bar{h}}_{nf}\text{(th)}$: Theoretical average nanofluid heat transfer coefficient (W·m^–^^2^·K^–^^1^)
*k*: Thermal conductivity (W·m^–1^·K^–1^)
*L*: Duct length (m)
$\bar{Nu}(\exp)$: Average nanofluid Nussult number obtained from experiments
$\bar{Nu}(\mathrm{sim})$: Average nanofluid Nussult number calculated from CFD analysis
*Pe*: Peclet number
*Pr*: Prandtl number
*q*: Heat flux (W/m^2^)
*Re*: Reynolds number
*T_b_*: Bulk temperature (K)
*T_w_*: Duct wall temperature (K)
$\bar{U}$: Average fluid velocity (m·s^–1^)
*u*: The x-component of the velocity (m·s^–^^1^)
*v*: The y-component of the velocity (m·s^–^^1^)
*w*: The z-component of the velocity (m·s^–^^1^)

### Greek Symbols

*γ*: Ratio of the nano-layer thickness to original particle radius
*μ*: Viscosity (Pa·s)
*μ_wnf_*: Nanofluid viscosity at duct wall temperature (Pa·s)
*ϕ*: Nanoparticle volume fraction (%)
*ρ*: Density (kg·m^–^^3^)

### Subscripts

bf: Base fluid
i: Inlet
nf: Nanofluid
o: Outlet
p: Solid nanoparticles
w: Wall
